# Undernutrition and Obesity Are Associated with Adverse Clinical Outcomes in Hospitalized Children and Adolescents with Acute Pancreatitis

**DOI:** 10.3390/nu13010043

**Published:** 2020-12-25

**Authors:** Aravind Thavamani, Krishna Kishore Umapathi, Thomas J. Sferra, Senthilkumar Sankararaman

**Affiliations:** 1 Department of Pediatrics (Divison of Pediatric Gastroenterology), UH Rainbow Babies and Children’s Hospital, Cleveland, OH 44106, USA; Aravind.thavamani@uhhospitals.org (A.T.); thomas.sferra@uhhospitals.org (T.J.S.); 2 Department of Pediatrics, Rush University Medical Center, Chicago, IL 60612, USA; krishnakishoreumapathi@gmail.com

**Keywords:** acute pancreatitis, pediatric, severe acute pancreatitis, malnutrition, undernutrition, obesity, nutritional status, health care utilization, outcomes

## Abstract

**Background:** Adult studies demonstrated that extremes of nutritional status adversely impact clinical outcomes in acute pancreatitis (AP). With rising prevalence of undernutrition/obesity in children, we analyzed the effect of nutritional status on the clinical outcomes in children and adolescents with acute pancreatitis. **Methodology:** We analyzed the Kids’ Inpatient Database (KID) between 2003 and 2016 to include all patients with a primary diagnosis of AP using specific International Classification of Diseases (ICD) codes. We classified into (1) undernutrition, (2) obesity and (3) control groups, based on ICD codes, and we compared severe acute pancreatitis and healthcare utilization (length of stay and hospitalization costs). **Results:** Total number of AP admissions was 39,805. The prevalence of severe AP was higher in the undernutrition and obesity groups than the control group (15.7% vs. 5.8% vs. 3.5% respectively, *p* < 0.001). Multivariate analyses demonstrated that undernutrition and obesity were associated with 2.5 and 1.6 times increased risk of severe AP, *p* < 0.001. Undernutrition was associated with an additional six days of hospitalization and almost $16,000 in hospitalization costs. Obesity was associated with an additional 0.5 day and almost $2000 in hospitalization costs, *p* < 0.001. **Conclusion:** Undernutrition and obesity were associated with greater severity of AP, as well as prolonged hospitalization stay and costs. It is imperative for treating clinicians to be aware of these high-risk groups to tailor management and strive for improved outcomes.

## 1. Introduction

Recent studies indicate that the incidence of acute pancreatitis (AP) is steadily increasing in children [1,2,3,4,5,6]. The incidence rate of AP-related hospitalization is even higher, and it increased from 10.5 per 10,000 in 2003 to 14.6 per 10,000 hospitalizations in 2016 [6]. The majority of cases of acute pancreatitis in children resolve spontaneously requiring supportive care, and, unlike in adults, the mortality rate of acute pancreatitis is low [6]. However, 4–34% of children and adolescents with acute pancreatitis may develop severe disease with complications requiring intensive care unit (ICU) admission and prolonged hospital stay, resulting in increased health care expenditure [6,7,8,9].

Even though malnutrition is often used to refer to undernutrition, in a strict sense, it refers to both undernutrition and overnutrition. Extremes of nutritional status is shown to adversely impact the outcome of many disease states in children [10,11,12]. Prior literature demonstrated that malnutrition in hospitalized children is associated with delayed recovery, prolonged hospitalization, and increased morbidity and mortality in children [13,14]. Studies in adults have shown that extremes of nutritional status (both undernutrition and obesity) are associated with adverse clinical outcomes and mortality among patients with AP [15,16]. In a recent meta-analysis, obesity has been recognized as an independent risk factor for organ failure in adults with AP [17]. Similarly, single-center pediatric studies have suggested that nutritional status might influence the severity of AP and may be associated with prolonged hospital stay [18,19,20]. A study from Japan utilizing an administrative database noted that children with obesity, when compared to children with normal nutritional status, had a prolonged hospital stay (25 days vs. 15 days) and incurred higher hospitalization costs (14,169 vs. 7457 USD) [19]. In our prior study, we demonstrated that morbid obesity had high prevalence of severe acute pancreatitis (SAP), increased length of stay, and higher hospital costs [6]. A larger, national-level, longitudinal study evaluating the impact of both undernutrition and obesity in children with AP is lacking. With changing epidemiology and both undernutrition and obesity being increasingly prevalent in the pediatric population, we sought to evaluate the impact of nutritional status on clinical outcomes and health care utilization in a larger cohort of children admitted with AP using a large, national-level administrative database [21,22].

## 2. Materials and Methods

We analyzed data from the Healthcare Cost and Utilization Project Kids’ Inpatient Database (HCUP-KID). The KID is a collection of data of hospitalized patients up to 20 years of age across the United States and has an aggregated collection of stratified random collection of data sampled at a rate of 80%. We analyzed the latest five KID datasets from 2003 to 2016 to include all inpatients encounters having AP as the primary diagnosis. Patients with chronic pancreatitis and those with secondary diagnosis of AP were excluded from the study. Each record analyzed in the datasets characterizes a discharge encounter after hospitalization. Each record contains a primary diagnosis for that pertinent hospitalization and up to 14 additional secondary concomitant diagnoses along with various procedural codes. All diagnostic and procedural codes were classified according to the International Classification of Diseases (ICD)-9-CM excluding KID 2016 that utilized ICD–10-CM codes for diagnosis and coding.

### 2.1. Variables and Outcomes

We included all patients up to 20 years of age having either ICD 9 (577.0) or ICD 10 diagnostic codes (K85) representing AP in the primary diagnosis column. Patients were then classified based on nutritional status into three mutually exclusive groups: (i) undernutrition (failure to thrive, body mass index (BMI) less than 5th centile, malnutrition diagnoses, marasmus or kwashiorkor), (ii) obesity (BMI ≥ 95th centile, BMI ≥ 30 kg/m^2^), and (iii) control, which included patients without the undernutrition or obesity codes. The list of ICD codes of the extracted diagnoses is listed in Supplementary Table S1.

We analyzed various demographic data including age of the patient, sex, insurance type, race, type of hospitalization, and hospital bed size. Race was classified into Caucasian, Hispanic, African American (AA), and the rest were categorized as other. Insurance was grouped into public insurance if patients had Medicare or Medicaid insurance, private (insurance/health maintenance organization), and the rest of the patients were categorized to be uninsured/self-pay, no charges, or others.

In our cohort of the AP population, we then analyzed for the presence of various etiologic and/or comorbid conditions including biliary disease (cholangitis, cholelithiasis), inflammatory bowel disease, diabetic ketoacidosis, alcohol-related pancreatitis, hypercalcemia, hypertriglyceridemia, anomalies of the pancreas, cystic fibrosis, systemic lupus erythematosus, solid organ transplants, abdominal trauma, and various common pediatric cancers.

The primary outcome of this study was the development of SAP defined according to the revised Atlanta classification, which requires the presence of end-organ damage including intubation/mechanical ventilation, or a concomitant presence of one of the following diagnoses of end-organ damage: acute kidney injury, pulmonary insufficiency, use of vasopressors, or systemic inflammatory response syndrome (SIRS) with end-organ dysfunction [23]. We then analyzed for secondary outcomes including the duration of hospital stay in days and total hospitalization costs to evaluate the healthcare resource utilization. Total costs were extracted from hospitalization charges using the cost-to-charge ratio data available in the KID datasets. We also compared various AP-related complications: pseudocyst of the pancreas, pulmonary embolism, deep vein thrombosis, and mortality rate between the groups. We also compared various procedures such as cholecystectomy, percutaneous/open biliary procedures, endoscopic retrograde cholangiopancreatography (ERCP), central venous catheterization, and pancreatectomy between the three defined groups based on their nutritional status.

### 2.2. Statistical Analysis

Discharge weights provided by the KID were used in the calculation of national estimates. As per the Hospitalization Cost and Utilization Project (HCUP) data user agreement, any value of less than 10 patient encounters was not reported to protect patient information and are represented as ≤10 in the tables. Continuous variables are reported as mean ± standard deviation, and categorical variables are reported as frequencies and percentages. Analysis of Variance (ANOVA) was used to compare continuous data, and Pearson’s χ^2^ test was used to compare categorical data. Logistic regression analysis was used to calculate adjusted odds ratio (OR) and confidence intervals (CIs) for various demographics and etiologic factors associated with severe AP. Multivariate linear regression models were constructed with length of stay and total hospitalization costs as outcome variables. Least-squares regression analysis was used to analyze the trend of nutritional status and acute pancreatitis over time. All analyses were performed on weighted data via SPSS 24. A *p* value < 0.05 was considered significant.

## 3. Results

**Figure 1:**
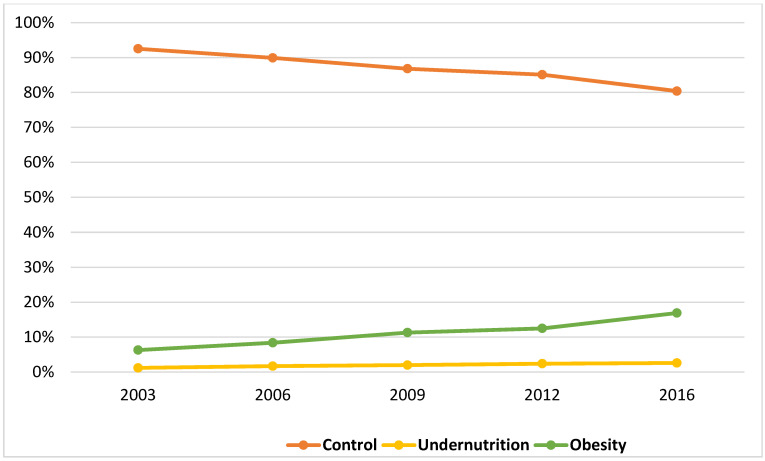
Increasing proportion of undernutrition and obesity among pediatric population with acute pancreatitis between 2003 and 2016.

We analyzed a total of 39,805 AP-related pediatric hospitalizations within the KID from 2003 to 2016. Based on the diagnostic codes, the prevalence of undernutrition and obesity were 2% and 11%, respectively. The combined overall prevalence of extremes of nutrition status (undernutrition and obesity) increased from 7.5% in 2003 to 19.6% in 2016, *p* < 0.001 (Figure 1). The prevalence of undernutrition increased from 1.2% in 2003 to 2.6% in 2016, *p* < 0.001. Similarly, the prevalence of obesity increased significantly by 2.7-fold, from 6.3% in 2003 to 16.9% in 2016, *p* < 0.001.

AP patients with undernutrition were younger (13.9 ± 5.9 years) and those with obesity were older (16.3 ± 3.3 years) as compared to the control group (15.1 ± 4.8 years), *p* < 0.001. The proportion of females was lowest in the undernutrition (54.7%) and highest in the obesity group (68.7) (Table 1). In the undernutrition group, the proportion of African Americans (AA) was higher than control population (13.6% vs. 10%), whereas in the obesity group, the relative proportion of Hispanics was higher (36.1% vs. 22.1%). The majority of patients with undernutrition and obesity had public insurance. Patients in the undernutrition group with AP were more often hospitalized in teaching hospitals compared to the obesity and control populations.

As expected, the prevalence of cholelithiasis was higher in the obesity group (43.7%) and less in the undernutrition (12.5%) and control groups (22.5%). Patients with obesity had increased prevalence of hypertriglyceridemia (8.5%), whereas hypercalcemia was more prevalent in the undernutrition group (1.2%). Overall, the undernutrition group had a greater number of co-existing chronic systemic conditions as described in Table 1.

A total of 1596 patients had SAP, accounting for 4% of the total population with AP. The prevalence rate was significantly higher within the undernutrition group (15.7%), followed by the obesity (5.8%) and control groups (3.5%), *p* < 0.001. (Table 2) Patients with undernutrition had increased prevalence of respiratory failure (9.6%), the use of mechanical ventilation (3.8%), and acute kidney injury (7.9%). Further, patients in the undernutrition group required increased utilization of parenteral nutrition in about 31% of cases. They also had increased risk of thrombotic complications, deep vein thrombosis/pulmonary embolism (undernutrition 1.9% vs. obesity 0.5% vs. control 0.2%, *p* < 0.001), increased rate of sepsis (undernutrition 7.9% vs. obesity 1.2% vs. control 1%, *p* < 0.001), and pseudocyst formation (undernutrition 11.7% vs. obesity 4.6% vs. control 2.7%, *p* < 0.001). Patients with obesity underwent more cholecystectomy and ERCP procedures, while the undernutrition group had more pancreatectomy procedures. Mortality rate was overall low among the pediatric population with AP (0.15%). (Table 2).

Multivariate logistic regression revealed that undernutrition was independently associated with 2.55 times (CI: 2.03 to 3.20, *p* < 0.001) increased odds of SAP, and obesity was associated with 1.6 times (CI:1.39 to 1.89, *p* < 0.001) increased odds of SAP compared to the control group (Table 3). Multivariate linear regression analysis showed that the undernutrition group was associated with 6 additional days (CI: 5.67 to 6.57, *p* < 0.001) of hospitalization and incurred almost $16,000 (CI: 14,317 to 17,520, *p* < 0.001) in additional hospitalization costs. Similarly, obesity was associated with 0.5 (CI: 0.32 to 0.73, *p* < 0.001) additional days of hospitalization and close to $2000 (CI: 1237 to 2667) in additional hospitalization costs compared to the control group (Table 3). Refer to Tables S2 and S3 in the supplement for the regression models.

## 4. Discussion

We present the largest study evaluating the impact of nutritional status in pediatric patients hospitalized with a primary diagnosis of AP. Further, we demonstrate that both undernutrition and obesity were associated with adverse clinical outcomes, specifically with the increased prevalence of SAP. We report an overall prevalence of SAP of 4%. In our cohort, after adjusting comorbid variables, SAP was increasingly associated 2.5 times and 1.6 times in undernutrition and obesity groups, respectively. Adults may develop severe complications such as end-organ failure and/or pancreatic necrosis from AP, and the resulting mortality in this population may be as high as 30% [24,25,26]. Unlike adults, children with AP usually have a mild clinical course with lower mortality, and the prevalence rate of SAP is usually lower [8,27,28,29,30]. However, few pediatric studies reported the prevalence rate of SAP to be as high as 34% [7,8,9,31]. Various reasons may account for this discrepancy, the main one being the utility of different criteria by various studies over the years. Specifically, in our cohort, several factors might have underestimated the prevalence rate of SAP. We excluded patients with a secondary diagnosis of AP who are generally sicker with increased mortality and morbidity [32]. The complications of SAP such as necrotizing pancreatitis and infected pancreatic tissue were not included due to lack of specific ICD codes. We also did not include pancreatic pseudocyst and pancreatic ascites in the diagnostic criteria as they may develop up to four weeks after AP and thus may not represent the acuity of the current episode of pancreatitis leading to hospitalization.

We report a mortality rate of 0.15% in the study population, which is significantly lower in comparison to adult patients with AP [33]. However, it is worth noting that 73% of the in-hospital mortality in our study cohort was associated with SAP, and the mortality rate among patients with SAP was 3.1%. Goday and colleagues, utilizing a multicenter database, evaluated 2076 children admitted to pediatric intensive care units (PICUs) with AP over a four-year period [32], most of whom (82%) had AP as a secondary diagnosis. The patients with secondary AP were sicker on PICU admission. The mortality was 0.3% in the primary AP group, which is comparable to our cohort, and in the secondary AP, mortality was higher at 6.8%. As our focus was to evaluate the impact of nutritional status in children admitted with AP, we excluded patients with a secondary diagnosis of AP who in general will be sicker with increased mortality, and this may explain the prevalence of lower mortality in our study population.

Vasilescu et al. in their single-center retrospective study showed that extremes of nutritional status may influence the severity of AP in pediatric patients [18]. They found that the length of stay was significantly longer for patients with undernutrition (16.5 days) compared to the patients with obesity (10.7 days) and control population (10.6 days). After adjusting comorbid conditions, we found undernutrition significantly affected AP patients with 6 additional days of hospitalization, and incurred almost $16,000 in additional hospitalization costs. We also demonstrated that the undernutrition group had increased disease burden with higher prevalence of various chronic inflammatory comorbid conditions such as systemic lupus erythematosus, inflammatory bowel disease, cystic fibrosis, and various malignancies. We hypothesize that the presence of multiple comorbid conditions and lack of energy reserve during periods of stress and catabolism linked to AP may predispose them to severe disease course. Although patients with undernutrition contributed only to a minority of AP patients, they represent the severe disease spectrum, warranting intensive care management to prevent morbidity and complications related to acute pancreatitis.

Despite obesity being increasingly prevalent, there are few studies evaluating the impact of obesity in pediatric patients with AP [21,34]. Studies have shown that patients with obesity have increased fat deposition in the pancreatic tissue [35,36,37,38]. These fat deposits could lead to necrosis during an episode of AP resulting in widespread necrosis and systemic release of inflammatory cytokines leading to end-organ damage [36,39]. Utilizing similar methodology, we previously demonstrated that AP children with morbid obesity had high prevalence of SAP (7.3% vs. 3.8% in the control population, *p* < 0.001), increased length of stay, and higher hospital costs [6]. In the current cohort, we found that the obesity group (pediatric patients with both obesity and morbid obesity) had longer length of hospitalization (5.4 vs. 4.8 days) and almost $2000 additional hospitalization costs. Cholelithiasis and hypertriglyceridemia were frequently associated with AP. Despite increasing incidence of AP in the pediatric population, well-validated prognostic scores to predict the severity of disease are still lacking [27]. Various prognostic scores included temporal laboratory values and imaging data to predict severity of AP and do not include nutritional status in their predictive model [7,8,9,29]. The pediatric acute pancreatitis score (PAPS) by DeBanto et al. included weight (<23 kg) as one of the predictors of severe disease [31]. Further prospective research evaluating the relationship of nutritional status on acute pancreatitis in pediatric patients is needed.

## 5. Limitations

Our study has limitations, including the retrospective nature of the analyses and limitations inherently applicable to any large administrative database analysis. The diagnostic accuracy depends on the coding correctness, and errors in coding should be taken into account while interpreting the results. As mentioned earlier, we were unable to analyze the data on pancreatic necrosis due to lack of specific ICD codes. We did not analyze morbid obesity as a separate group, which was previously shown with worse outcomes [6,15]. We did not have data on longitudinal follow up, laboratory values, and medication use, which are further limitations. We could not incorporate data on the enteral nutrition, which could potentially influence the clinical course of pancreatitis and prevention of development of complications. Although we did not have absolute BMI values and body composition data for each patient, there exists specific codes to define undernutrition and obesity, which are validated in prior studies using administrative databases, thus helping us to state our findings with reasonable confidence [40,41,42].

Despite these limitations, we demonstrated a significant association between nutritional status and adverse hospital outcomes in pediatric patients admitted with AP. The strengths of this study include the sample size of almost 40,000 AP-related hospitalizations and a nationally representative sample; this helped us to account for various comorbid conditions and complications related to AP in children.

## 6. Conclusions

The prevalence of undernutrition and obesity among pediatric patients admitted with AP has steadily increased between 2003 and 2016. In 2016, almost 1 in 5 children with AP had either undernutrition or obesity and was associated with increasing severity of the disease. Recognition of these patients at risk of severe disease course is crucial to improve clinical outcomes. Further well-designed longitudinal studies are needed to better understand the relationship between nutrition status and clinical outcomes in hospitalized children and adolescents with AP.

## Figures and Tables

**Table 1:** Comparison of various demographics, hospital factors, etiologies, and associated comorbid diseases of acute pancreatitis. Patients are classified based on nutritional status: Kids’ Inpatient Database 2003, 2006, 2009, 2012, and 2016.

Parameters	Control	Undernutrition	Obesity	*p* Value
	Group	Group	Group	
	*n* = 34,660	*n* = 782	*n* = 4363	
Demographics				
Age in years (Mean ± SD)	15.1 ± 4.8	13.9 ± 5.9	16.3 ± 3.3	<0.001
Gender	-	-	-	<0.001
Male	14,350 (41.9%)	354 (45.3%)	1363 (31.3%)	
Female	19,867 (58.9%)	428 (54.7%)	2992 (68.7%)	
Race	-	-	-	<0.001
Caucasian	15,402 (44.4%)	337 (43.1%)	1545 (35.4%)	
African American	3460 (10%)	106 (13.6%)	456 (10.4%)	
Hispanic	7674 (22.1%)	156 (19.9%)	1575 (36.1%)	
Others	8123 (23.4%)	183 (23.4%)	788 (18.1%)	
Insurance	-	-	-	<0.001
Public	13,953 (40.3%)	336 (43%)	2278 (52.2%)	
Private	15,837 (45.7%)	330 (42.3%)	1474 (33.8%)	
Self-pay/Other/Uninsured	4870 (14.1%)	115 (14.7%)	611 (14%)	
Location/Teaching Status	-	-	-	<0.001
Rural	3922 (11.6%)	36 (4.8%)	345 (8.1%)	
Urban Non-Teaching	10,482 (31.1%)	171 (22.8%)	1419 (33.1%)	
Urban Teaching	19,329 (57.3%)	544 (72.4%)	2518 (58.8%)	
Hospitalization	-	-	-	<0.01
Elective	2250 (6.5%)	55 (7.1%)	228 (5.2%)	
Non-Elective	32,288 (93.5%)	724 (92.9%)	4123 (94.8%)	
Etiologic and Comorbid Conditions				
Acute cholangitis	223 (0.6%)	17 (2.2%)	37 (0.8%)	<0.001
Cholelithiasis	7788 (22.5%)	98 (12.5%)	1908 (43.7%)	<0.001
Other Biliary Diseases	1088 (3.1%)	46 (5.9%)	163 (3.7%)	<0.001
Hypertriglyceridemia	737 (2.1%)	31 (4%)	369 (8.5%)	<0.001
Hypercalcemia	111 (0.3%)	10(1.2%)	10 (0.2%)	<0.001
Abdominal trauma	122 (0.4%)	≤10	≤10	<0.001
Anomalies of pancreas	202 (0.6%)	≤10	≤10	<0.001
Diabetic ketoacidosis	704 (2%)	20 (2.6%)	121 (2.8%)	0.004
Systemic Lupus Erythematosus	181 (0.5%)	14 (1.8%)	12 (0.3%)	<0.001
Cystic Fibrosis	295 (0.9%)	14 (1.8%)	≤10	<0.001
Inflammatory Bowel Disease	815 (2.4%)	29 (3.7%)	18 (0.4%)	<0.001
Malignancies	872 (2.5%)	60 (7.7%)	25 (0.6%)	<0.001
Solid Organ Transplant	217 (0.6%)	11 (1.4%)	≤10	<0.001
Alcohol Related	1846 (5.3%)	34 (4.4%)	120 (2.8%)	<0.001

**Table 2:** Comparison of surgical interventions and clinical outcome measures in acute pancreatitis. Patients are stratified based on nutritional status: Kids’ Inpatient Database 2003, 2006, 2009, 2012 and 2016.

Parameters	Control	Undernutrition	Obesity	*p* Value
	Group	Group	Group	
	*n* = 34,660	*n* = 782	*n* = 4363	
Outcomes				
Severe acute pancreatitis	1219 (3.5%)	123 (15.7%)	254 (5.8%)	<0.001
Length of stay (Mean ± SE) in days	4.8 ± 0.02	14.1 ± 0.73	5.4 ± 0.09	<0.001
Total hospital costs (Mean ± SE in USD)	9327 ± 107	34,089 ± 3339	11,827 ± 295	<0.001
Mortality	55 (0.2%)	≤10	≤10	0.97
AKI	465 (1.3%)	62 (7.9%)	79 (1.8%)	<0.001
Respiratory Failure	729 (2.1%)	75 (9.6%)	178 (4.1%)	<0.001
Invasive Mechanical Ventilation	339 (1%)	30 (3.8%)	56 (1.3%)	<0.001
SIRS with organ dysfunction	23 (0.1%)	≤10	10 (0.2%)	0.002
Use of Vasopressors	18 (0.1%)	≤10	≤10	<0.001
CVC placement	1959 (5.7%)	198 (25.3%)	245 (5.6%)	<0.001
Parenteral Nutrition	1967 (5.7%)	241 (30.9%)	243 (5.6%)	<0.001
Sepsis	341 (1%)	62 (7.9%)	54 (1.2%)	<0.001
PE/DVT	46 (0.2%)	15 (1.9%)	23 (0.5%)	<0.001
Pseudocyst	953 (2.7%)	91 (11.7%)	199 (4.6%)	<0.001
Cholecystectomy	4773 (13.8%)	52 (6.6%)	1063 (24.4%)	<0.001
Any ERCP	2521 (7.3%)	55 (7%)	472 (10.8%)	<0.001
Percutaneous/Open Biliary Procedures	151 (0.5%)	≤10	≤10	0.08
Pancreatectomy	144 (0.4%)	19 (2.4%)	27 (0.6%)	<0.001

AKI—acute kidney injury; SIRS—systemic inflammatory response syndrome; CVC—central venous catheter, PE—pulmonary embolism, DVT—deep vein thrombosis, ERCP—endoscopic retrograde cholangiopancreatography. USD—United States Dollar.

**Table 3:** Multivariate analysis evaluating the impact of nutritional status on the outcomes of severe acute pancreatitis, length of stay, and total hospitalization costs among all patients with acute pancreatitis.

Outcomes	Odds ratio	95% CI	*p* value
Severe acute pancreatitis *			
Control group	Reference	-	-
Undernutrition group vs. Control group	2.55	2.03 to 3.20	<0.001
Obesity group vs. Control group	1.62	1.39 to 1.89	<0.001
	**Average Difference**	**95% CI**	***p* value**
Length of stay (in days) **			
Control group	Reference		
Undernutrition group vs. Control group	6.12	5.67 to 6.57	<0.001
Obesity group vs. Control group	0.53	0.32 to 0.73	<0.001
Total hospitalization costs (in USD) **			
Control group	Reference	-	-
Undernutrition group vs. Control group	15,919	14,317 to 17,520	<0.001
Obesity group vs. Control group	1952	1237 to 2667	<0.001

USD—United States dollar. * Please refer to supplemental Table S2 for the multiple logistic regression model with severe acute pancreatitis as outcome variables. ** Please refer to supplemental Table S3 for the multiple linear regression models with length of stay and total hospitalization costs as outcome variables.

## Data Availability

No new data were created or analyzed in this study. Data sharing is not applicable to this article.

## Supplementary Materials

The following are available online at https://www.mdpi.com/2072-6643/13/1/43/s1, Supplementary Table S1 List of diagnostic and procedural codes utilized using the International Classification of Disease (ICD)-9-CM for Kids’ Inpatient Database 2003, 2006, 2009, and 2012 and ICD–10-CM for Kids’ Inpatient Database 2016. Supplementary Table S2. Multiple logistic regression model of various factors associated with severe acute pancreatitis. Supplementary Table S3 Multiple linear regression model of various factors associated with length of stay and total hospitalization costs.

## References

[1] Hornung L., Szabo F.K., Kalkwarf H.J., Abu-El-Haija M. (2017). Increased Burden of Pediatric Acute Pancreatitis on the Health Care System. Pancreas.

[2] Cheng Y.J., Yang H.Y., Tsai C.F., Lin J.S., Lee H.C., Yeung C.Y., Chen S.C. (2019). Epidemiology of Pediatric Acute Pancreatitis in Taiwan: A Nationwide Population-based Study. J. Pediatric Gastroenterol. Nutr..

[3] Nydegger A., Heine R.G., Ranuh R., Gegati-Levy R., Crameri J., Oliver M.R. (2007). Changing incidence of acute pancreatitis: 10-year experience at the Royal Children’s Hospital, Melbourne. J. Gastroenterol. Hepatol..

[4] Morinville V.D., Barmada M.M., Lowe M.E. (2010). Increasing incidence of acute pancreatitis at an American pediatric tertiary care center: Is greater awareness among physicians responsible?. Pancreas.

[5] Pant C., Deshpande A., Olyaee M., Anderson M.P., Bitar A., Steele M.I., Bass P.F., Sferra T.J. (2014). Epidemiology of acute pancreatitis in hospitalized children in the United States from 2000–2009. PLoS ONE..

[6] Thavamani A., Umapathi K.K., Roy A., Krishna S.G. (2020). The increasing prevalence and adverse impact of morbid obesity in paediatric acute pancreatitis. Pediatr. Obes..

[7] Szabo F.K., Hornung L., Oparaji J.A., Alhosh R., Husain S.Z., Liu Q.Y., Palermo J., Lin T.K., Nathan J.D., Podberesky D.J. (2016). A prognostic tool to predict severe acute pancreatitis in pediatrics. Pancreatology.

[8] Lautz T.B., Chin A.C., Radhakrishnan J. (2011). Acute pancreatitis in children: Spectrum of disease and predictors of severity. J. Pediatr. Surg..

[9] Coffey M.J., Nightingale S., Ooi C.Y. (2013). Serum lipase as an early predictor of severity in pediatric acute pancreatitis. J. Pediatr. Gastroenterol. Nutr..

[10] Irving S.Y., Daly B., Verger J., Typpo K.V., Brown A.M., Hanlon A., Weiss S.L., Fitzgerald J.C., Nadkarni V.M., Thomas N.J. (2018). The association of nutrition status expressed as body mass index z-score with outcomes in children with severe sepsis: A secondary analysis from the Sepsis Prevalence, Outcomes and Therapies (SPROUT) study. Crit. Care Med..

[11] De Souza Menezes F., Leite H.P., Nogueira P.C.K. (2012). Malnutrition as an independent predictor of clinical outcome in critically ill children. Nutrition.

[12] Orgel E., Genkinger J.M., Aggarwal D., Sung L., Nieder M., Ladas E.J. (2016). Association of body mass index and survival in pediatric leukemia: A meta-analysis. Am. J. Clin. Nutr..

[13] Daskalou E., Galli-Tsinopoulou A., Karagiozoglou-Lampoudi T., Augoustides-Savvopoulou P. (2016). Malnutrition in hospitalized pediatric patients: Assessment, prevalence, and association to adverse outcomes. J. Am. Coll. Nutr..

[14] Caulfield L.E., de Onis M., Blössner M., Black R.E. (2004). Undernutrition as an underlying cause of child deaths associated with diarrhea, pneumonia, malaria, and measles. Am. J. Clin. Nutr..

[15] Krishna S.G., Hinton A., Oza V., Hart P.A., Swei E., El-Dika S., Stanich P.P., Hussan H., Zhang C., Conwell D.L. (2015). Morbid obesity is associated with adverse clinical outcomes in acute pancreatitis: A propensity-matched study. Am. J. Gastroenterol..

[16] Kim Y.J., Kim D.B., Chung W.C., Lee J.M., Youn G.J., Jung Y.D., Choi S., Oh J.H. (2017). Analysis of factors influencing survival in patients with severe acute pancreatitis. Scand. J. Gastroenterol..

[17] Smeets X.J., Knoester I., Grooteman K.V., Singh V.K., Banks P.A., Papachristou G.I., Duarte-Rojo A., Robles-Diaz G., Kievit W., Besselink M.G. (2019). The association between obesity and outcomes in acute pancreatitis: An individual patient data meta-analysis. Eur. J. Gastroenterol. Hepatol..

[18] Vasilescu A., Cuffari C., Santo Domingo L., Scheimann A.O. (2015). Predictors of severity in childhood pancreatitis: Correlation with nutritional status and racial demographics. Pancreas.

[19] Murata A., Ohtani M., Muramatsu K., Kobori S., Tomioka S., Matsuda S. (2016). Impact of obesity on outcomes of paediatric acute pancreatitis based on a national administrative database. Pediatr Obes..

[20] Kittikundecha T., Lakananurak N., Rerknimitr R. (2019). Using Nutrition Risk Scores to Predict Hospital Length of Stay in Mild Acute Pancreatitis: A Prospective Cohort Study. J. Med. Assoc. Thail..

[21] Skinner A.C., Ravanbakht S.N., Skelton J.A., Perrin E.M., Armstrong S.C. (2018). Prevalence of obesity and severe obesity in US children, 1999–2016. Pediatrics.

[22] Carvalho-Salemi J., Salemi J.L., Wong-Vega M.R., Spooner K.K., Juarez M.D., Beer S.S., Canada N.L. (2018). Malnutrition among hospitalized children in the United States: Changing prevalence, clinical correlates, and practice patterns between 2002 and 2011. J. Acad. Nutr. Diet..

[23] Banks P.A., Bollen T.L., Dervenis C., Gooszen H.G., Johnson C.D., Sarr M.G., Tsiotos G.G., Vege S.S. (2013). Classification of acute pancreatitis—2012: Revision of the Atlanta classification and definitions by international consensus. Gut.

[24] Petrov M.S., Shanbhag S., Chakraborty M., Phillips A.R., Windsor J.A. (2010). Organ failure and infection of pancreatic necrosis as determinants of mortality in patients with acute pancreatitis. Gastroenterology.

[25] Garg P.K., Madan K., Pande G.K., Khanna S., Sathyanarayan G., Bohidar N.P., Tandon R.K. (2005). Association of extent and infection of pancreatic necrosis with organ failure and death in acute necrotizing pancreatitis. Clin. Gastroenterol. Hepatol..

[26] Werge M., Novovic S., Schmidt P.N., Gluud L.L. (2016). Infection increases mortality in necrotizing pancreatitis: A systematic review and meta-analysis. Pancreatology.

[27] Abu-El-Haija M., Kumar S., Quiros J.A., Balakrishnan K., Barth B., Bitton S., Eisses J.F., Foglio E.J., Fox V., Francis D. (2018). The management of acute pancreatitis in the pediatric population: A clinical report from the NASPGHAN Pancreas Committee. J. Pediatric Gastroenterol. Nutr..

[28] Suzuki M., Saito N., Naritaka N., Nakano S., Minowa K., Honda Y., Ohtsuka Y., Yamataka A., Shimizu T. (2015). Scoring system for the prediction of severe acute pancreatitis in children. Pediatrics Int..

[29] Bierma M.J., Coffey M.J., Nightingale S., van Rheenen P.F., Ooi C.Y. (2016). Predicting severe acute pancreatitis in children based on serum lipase and calcium: A multicentre retrospective cohort study. Pancreatology.

[30] Werlin S.L., Kugathasan S., Frautschy B.C. (2003). Pancreatitis in children. J. Pediatr. Gastroenterol. Nutr..

[31] DeBanto J.R., Goday P.S., Pedroso M.R., Iftikhar R., Fazel A., Nayyar S., Conwell D.L., DeMeo M.T., Burton F.R., Whitcomb D.C. (2002). Acute pancreatitis in children. Am. J. Gastroenterol..

[32] Goday P.S., Wakeham M., Kuhn E.M., Collins M.M., Werlin S.L. (2015). Acute pancreatitis in the pediatric intensive care unit. J. Pediatr. Gastroenterol. Nutr..

[33] Gapp J., Hall A.G., Walters R.W., Jahann D., Kassim T., Reddymasu S. (2019). Trends and outcomes of hospitalizations related to acute pancreatitis: Epidemiology from 2001 to 2014 in the United States. Pancreas.

[34] Skinner A.C., Skelton J.A. (2014). Prevalence and trends in obesity and severe obesity among children in the United States, 1999–2012. JAMA Pediatr..

[35] Acharya C., Navina S., Singh V.P. (2014). Role of pancreatic fat in the outcomes of pancreatitis. Pancreatology.

[36] Noel P., Patel K., Durgampudi C., Trivedi R.N., De Oliveira C., Crowell M.D., Pannala R., Lee K., Brand R., Chennat J. (2016). Peripancreatic fat necrosis worsens acute pancreatitis independent of pancreatic necrosis via unsaturated fatty acids increased in human pancreatic necrosis collections. Gut.

[37] Premkumar R., Phillips A.R., Petrov M.S., Windsor J.A. (2015). The clinical relevance of obesity in acute pancreatitis: Targeted systematic reviews. Pancreatology.

[38] Maggio A.B., Mueller P., Wacker J., Viallon M., Belli D.C., Beghetti M., Farpour-Lambert N.J., McLin V.A. (2012). Increased pancreatic fat fraction is present in obese adolescents with metabolic syndrome. J. Pediatr. Gastroenterol. Nutr..

[39] Khatua B., El-Kurdi B., Singh V.P. (2017). Obesity and pancreatitis. Curr. Opin. Gastroenterol..

[40] Abdelhadi R.A., Bouma S., Bairdain S., Wolff J., Legro A., Plogsted S., Guenter P., Resnick H., Slaughter-Acey J.C., Corkins M.R. (2016). Characteristics of hospitalized children with a diagnosis of malnutrition: United States, 2010. J. Parenter. Enter. Nutr..

[41] Zwintscher N.P., Horton J.D., Steele S.R. (2014). Obesity has minimal impact on clinical outcomes in children with inflammatory bowel disease. J. Pediatr. Surg..

[42] Okubo Y., Nochioka K., Hataya H., Sakakibara H., Terakawa T., Testa M. (2016). Burden of obesity on pediatric inpatients with acute asthma exacerbation in the United States. J. Allergy Clin. Immunol. Pract..

