# Supplementary material for: Undernutrition and Obesity Are Associated with Adverse Clinical Outcomes in Hospitalized Children and Adolescents with Acute Pancreatitis
Source: Nutrients. 2020 Dec 25;13(1):43. doi: 10.3390/nu13010043 (PMC7824217; doi:10.3390/nu13010043)
Supplement: Supplementary file 1 [file nutrients-13-00043-s001.pdf]

**Table S1.** List of diagnostic and procedural codes utilized using the International Classification of Disease (ICD)-9-CM for Kids' Inpatient Database 2003, 2006, 2009, and 2012 and ICD–10-CM for Kids' Inpatient Database 2016.

ICD codes:

| Diagnosis | ICD 9 | ICD 10 |
|---|---|---|
| Acute pancreatitis | 577.0 | K85* |
| Chronic pancreatitis | 577.1 | K86* |
| Pseudocyst of pancreas | 5772 | K86.3 |
| Cholelithiasis/choledocholithiasis | 574* | K80.* |
| Cholangitis | 576.1 | K83.* |
| Other biliary disease | 576.2, 576.8, 782.4, 576.9 | K83.1, K83.8, K83.9, R17 |
| IBD | 555*, 556* | K50*, K51* |
| Cystic Fibrosis | 277.0* | E84* |
| Hypertriglyceridemia | 272.1 | E78.1 |
| Hypercalcemia | 275.42 | E83.52 |
| Abdominal trauma | 863*, 864*, 865*, 866*, 867*, 868*, 869*. | S36 * to S39* |
| Anomalies of pancreas | 751.7 | Q45.0 to Q45.3 |
| DKA | 250.1* | E081, E0811, E091, E0911, E101, E1011, E111, E1111 |
| SLE | 695.4, 710.0 | M32*, L93* |
| Solid organ transplant | 996.8*, V42 to V42.7, V42.84, , V 42.83, Z94.83, V42.9 | T86.1*, T86.2*, T86.3*, T86.4* |
| Alcohol related | 291*, 303*, 305*, 760.71, 980.0, 357.5, 425.5, 535.30, 535.31, 571* | G62.1, G31.2, T510X1A, T510X2A, T510X3A, T510X4A, F10*, Y90*, K70*, Z714*, K29.0, K29.1, K29.2. |
| Pulmonary embolism | 415.1* | I26* |
| Deep Vein thrombosis | 453.4*, 453.82, 453.82 | I824*, I826* |
| Use of Vasopressor# | 0017 | 3E030XZ, 3E033XZ, 3E040XZ, 3E043XZ, 3E050XZ, 3E053XZ, 3E060XZ, 3E063XZ |
| Intubation and mechanical ventilation# | 93.90, 96.0*, 96.7* | 5A09357, 5A09457, 5A09557, 09HN7BZ, 0CHY7BZ, 0DH57BZ, 0NH17EZ, 5A1935Z, 5A1945Z, 5A1955Z, 0BH07DZ |
| Central venous catheter# | 38.93, 86.07 | 0JH63XZ, 0JHD3XZ, 0JHF3WZ, 0JHM3XZ, 0JHL3XZ |
| Common Bile duct exploration# | 51.02, 51.03, 51.04, 41.32, 51.36, 51.37, 51.39, 51.41, 51.43, 51.51, 51.59, 51.63, 51.64, 51.69, 51.71, 51.79 | 0FT90ZZ, 0FT50ZZ, 0FT60ZZ, 0FT70ZZ, 0FT80ZZ, 0F550ZZ, 0F560ZZ, 0F570ZZ, 0F580ZZ, 0FB50ZZ, 0FB60ZZ, 0FB70ZZ, 0FB80ZZ, 0FC90ZZ, 0F9900Z, 0F9900Z, 0F990ZZ, 0F9940Z, OF9400Z, 0FC400Z, 0FQ97ZZ, 0FQ90ZZ, 0F150D3, 0F150DB, 0F150Z3, 0F150ZB, 0F160D3, 0F160DB, 0F160Z3, 0F160ZB, 0F170D3, 0F170DB, 0F170Z3, 0F170ZB, 0F180D3, 0F180DB, 0F180Z3, 0F180ZB, 0F190D3, 0F190DB, 0F190Z3, 0F190ZB, 0F140D3, 0F140DB, 0F140Z3, 0F140ZB, |
| Cholecystectomy# | 51.2* | 0FB40ZX, 0FB40ZZ, 0FB43ZX, 0FB43ZZ, 0FB48ZX, 0FB48ZZ, 0FB44ZX, 0FB44ZZ, |
| Pancreatectomy# | 52.22, 52.95, 52.96 52.99 | 0F7D0ZZ, 0FQD0ZZ, 0FQG0ZZ, 0F1D0D3, 0F1D0DB, 0F1D0Z3, 0F1D0ZB, 0F1G0D3, 0F1G0ZB, 0F1G0ZZ, OFTG0ZZ, 0F1G0ZC, 0FTG0ZZ, 0FBG0ZZ, 0F9G00Z, 0FCD0ZZ, 0FCD8ZZ, 0F9D0ZX, 0F9D8ZZ |
| ERCP# | 51.83, 51.84, 51.85, 51.86, 51.87, 51.88, 51.10, 51.11, 51.14, 52.13, 52.93, 52.94, 52.98, 97.05. | 0FQC7ZZ, 0FQC8ZZ, 0F758ZZ, 0F768ZZ, 0F778ZZ, 0F7C8DZ, 0F7C8ZZ, 0F958ZZ, 0F968ZZ, 0F978ZZ, 0F988ZZ, 0F998ZZ, 0F9C8ZZ, 0F9D8ZZ, 0F9C80Z, 0F9980Z, 0F758DZ, 0F768DZ, 0F778DZ, 0F788DZ, 0F798DZ, 0FHB4DZ, 0FHB8DZ, 0FC58ZZ, 0FC68ZZ, 0FC78ZZ, 0FC98ZZ, 0FC88ZZ, 0FCC8ZZ, 0FF48ZZ, 0FF58ZZ, 0FF68ZZ, 0FF78ZZ, 0FF88ZZ, 0FF98ZZ, 0FFC8ZZ, 0F959ZX, 0F968ZX, 0F978ZX, 0F988ZX, 0F998ZX, 0F9C8ZX, 0FB48ZX, 0FB58ZX, 0FB68ZX, 0FB78ZX, 0FB88ZX, 0FB98ZX, 0FBC8ZX, 0FHD8DZ, 0FCD8ZZ, 0F7D8ZZ, 0F758DZ, 0F778DZ, 0F798DZ, 0F788DZ, 0F7D8DZ, 0FHB8DZ, 0FPB8DZ,0FPD8DZ |
| Percutaneous biliary procedures# | 51.01, 51.96, 51.98 | 0F943ZZ, 0FC93ZZ, 0F753Dz, 0F753DZ, 0F63ZZ, 0F783DZ, 0F783ZZ, 0F793ZZ, 0F793DZ, 0F993ZZ, 0F994ZZ, 0FC53ZZ, 0FC54ZZ, 0FC64ZZ, 0FC84ZZ, 0FC84ZZ, 0FC83ZZ, 0FC63ZZ. |
| Acute respiratory failure | 518.0, 518.81, 518.82, 518.84 | J96.0*, J96.2*, J98.1* |
| Acute Renal Failure | 584.* | N17* |
| Systemic inflammatory response syndrome | 995.94 | R65.10, R65.11 |
| Obesity | 278.00, 278.01, 278.03, V85.3, V85.4, V85.54 | E66.0, E66.1, E66.2, E66.8, E66.9, Z68.3, Z68.4, Z68.54 |
| Undernutrition | V85.51, V85.0, 260, 261, 262, 263 | Z68.51, Z68.1, E40, E41, E42, E43, E44, E45, E46 |

# Procedure codes.

**Table S2.** Multiple logistic regression model of various factors associated with severe acute pancreatitis.

| Parameters | OR (Confidence Interval) | P Value |
|---|---|---|
| Age | 1.02 (1.01 to 1.03) | <0.001 |
| Female vs Male | 0.68 (0.61 to 0.76) | <0.001 |
| Insurance | | |
| Public | Reference | |
| Private | 0.84 (0.74 to 0.94) | <0.001 |
| Others/Self Pay | 0.80 (0.68 to 0.96) | <0.001 |
| Race | | |
| Caucasian | Reference | |
| African American | 1.15 (0.96 to 1.37) | 0.10 |
| Hispanics | 0.96 (0.83 to 1.10) | 0.60 |
| Others | 0.94 (0.81 to 1.08) | 0.39 |
| Location/Teaching status | | |
| Rural | Reference | |
| Urban Non-teaching | 2.08 (1.58 to 2.73) | <0.001 |
| Urban Teaching | 2.72 (2.09 to 3.55) | <0.001 |
| Cholangitis | 2.56 (1.64 to 4.02) | <0.001 |
| Cholelithiasis | 0.91 (0.79 to 1.05) | 0.23 |
| Other Biliary Disease | 0.87 (0.64 to 1.16) | 0.35 |
| Hypertriglyceridemia | 1.95 (1.55 to 2.44) | <0.001 |
| Hypercalcemia | 5.26 (3.31 to 8.36) | <0.001 |
| Diabetic Ketoacidosis | 3.57 (2.85 to 4.48) | <0.001 |
| Systemic Lupus Erythematosus | 4.98 (3.4 to 7.30) | <0.001 |
| Cystic fibrosis | 0.69 (0.34 to 1.42) | 0.32 |
| Inflammatory Bowel Disease | 0.28 (0.15 to 0.52) | <0.001 |
| Malignancy | 2.46 (1.56 to 3.76) | <0.001 |
| Organ transplant | 2.42 (1.56 to 3.76) | <0.001 |
| Alcohol related | 1.03 (0.80 to 1.33) | 0.79 |
| Nutritional Status | | |
| Control population | Reference | |
| undernutrition | 2.55 (2.03 to 3.20) | <0.001 |
| Obesity | 1.62 (1.39 to 1.89) | <0.001 |

**Table S3.** Multiple linear regression model of various factors associated with length of stay and total hospitalization costs.

| Parameters | Length of stay (days) | | Total hospitalization costs (USD) | |
|---|---|---|---|---|
| | Days (CI) | P Value | USD (CI) | P value |
| Age | -0.04 (-0.06 to -0.03) | <0.001 | -99 (-151 to -47) | <0.001 |
| Female vs Male | 0.02 (-0.01 to 0.15) | 0.74 | -56 (-522 to 409) | 0.81 |
| Insurance | | | | |
| Private | Ref | | Ref | |
| Public | 0.29 (0.14 to 0.44) | <0.001 | 1138 (641 to 1635) | <0.001 |
| Others/Self Pay | -0.07 (-0.28 to 0.13) | 0.47 | -77 (-768 to 614) | 0.82 |
| Race | | | | |
| Caucasian | Ref | | Ref | |
| African American | 0.10 (-0.14 to 0.35) | 0.42 | 684 (-153 to 1523) | 0.10 |
| Hispanics | 0.08 (-0.11 to 0.28) | 0.41 | 1485 (816 to 2154) | <0.001 |
| Others | -0.06 (-0.24 to 0.10) | 0.44 | 378 (-194 to 951) | 0.19 |
| Location/Teaching status | | | | |
| Rural | Ref | | | |
| Urban Non-teaching | 1.21 (1.03 to 1.45) | 0.043 | 734 (534 to 1043) | <0.001 |
| Urban Teaching | 1.43 (1.22 to 1.58) | <0.001 | 2976 (1346 to 4412) | <0.001 |
| Cholangitis | 2.18 (1.44 to 2.93) | <0.001 | 5355 (2766 to 7943) | <0.001 |
| Cholelithiasis | 0.68 (0.52 to 0.84) | <0.001 | 3609 (3041 to 4176) | <0.001 |
| Other Biliary Disease | 1.06 (0.71 to 1.41) | <0.001 | 2545 (1325 to 3765) | <0.001 |
| Hypertriglyceridemia | 0.76 (0.37 to 1.15) | <0.001 | 1643 (297 to 2989) | <0.001 |
| Hypercalcemia | 1.65 (0.58 to 2.73) | 0.03 | 5300 (1614 to 8986) | <0.001 |
| Diabetic Ketoacidosis | 1.02 (0.58 to 1.46) | <0.001 | 3031 (1475 to 4588) | <0.001 |
| Systemic Lupus Erythematosus | 4.97 (4.12 to 5.83) | <0.001 | 9886 (6871 to 12901) | <0.001 |
| Cystic fibrosis | 0.11 (-0.58 to 0.81) | 0.74 | 789 (-1628 to 3207) | <0.001 |
| Inflammatory Bowel Disease | 0.01 (-0.4 to 0.44) | 0.94 | 547 (-962 to 2058) | <0.001 |
| Malignancy | 5.14 (4.73 to 5.55) | <0.001 | 22593 (21162 to 24042) | <0.001 |
| Organ transplant | 0.46 (-0.34 to 1.28) | 0.26 | 3783 (890 to 6675) | <0.001 |
| Alcohol related | 0.09 (-0.21 to 0.39) | <0.001 | 63 (-993 to 1120) | <0.001 |
| Nutritional Status | | | | |
| Control population | Reference | | Reference | |
| undernutrition | 6.12 (5.67 to 6.57) | <0.001 | 15,919 (14317 to 17520) | <0.001 |
| Obesity | 0.53 (0.32 to 0.73) | <0.001 | 1,952 (1237 to 2667) | <0.001 |
